# Health care policy trial of primary human papillomavirus–based cervical screening in Denmark: Comparison of three triage algorithms

**DOI:** 10.1002/ijc.70365

**Published:** 2026-02-07

**Authors:** Jeppe Bennekou Schroll, Jesper Bonde, Elsebeth Lynge, Marianne Waldstrøm, Petra Hall Viborg, Anna Frandsen, Rikke Holst Andersen, Susanne Merete Nielsen, Bettina Kjær Kristensen, Doris Schledermann, Berit Andersen, the National Danish Cervical Cancer Screening Steering Committee

**Affiliations:** ^1^ Cochrane Denmark & Centre for Evidence‐Based Medicine Odense (CEBMO), Department of Clinical Research University of Southern Denmark Odense Denmark; ^2^ Open Patient Data Explorative Network (OPEN) Odense University Hospital Odense Denmark; ^3^ Department of Gynecology & Obstetrics Herlev‐Gentofte Hospital Herlev Denmark; ^4^ Molecular Pathology Laboratory, Department of Pathology, afs. 134 AHH‐Hvidovre Hospital, Copenhagen University Hospital Hvidovre Denmark; ^5^ Department of Public Health University of Copenhagen Copenhagen Denmark; ^6^ Department of Pathology Aarhus University Hospital Aarhus Denmark; ^7^ Department of Clinical Medicine Aarhus University Aarhus Denmark; ^8^ The Danish Healthcare Quality Institute (DHQI) Rigshospitalet Copenhagen Denmark; ^9^ Department of Pathology Aalborg University Hospital Ålborg Denmark; ^10^ Department of Pathology Region Hospital Randers Randers Denmark; ^11^ Department of Pathology Region Zealand Næstved Denmark; ^12^ UNICCA, University Research Clinic for Cancer Screening, Department of Public Health Programmes Randers Regional Hospital Randers Denmark; ^13^ Department of Pathology Lille Baelt Hospital Vejle Denmark

**Keywords:** cervical cancer screening, dual stain, health care policy, HPV, HPV genotype, triage

## Abstract

Women 30–59 years were allocated to either HPV‐based screening or cytology‐based screening in this Danish health care policy trial. The optimal triage of HPV‐positive women could be a combination of cytology triage with HPV genotyping or p16/Ki67 staining. We report number of screen positives, colposcopies, and cervical lesions of three different triage algorithms (p16/Ki67, HPV16/18, or HPV16/18/31/33/52) in HPV‐positive women with low‐grade cytological abnormalities. We included 178,317 women with a sample in 2021 of which 91,517 were screened with HPV and 86,800 with cytology. All women were followed for 18 months. Almost three times as many women screened positive with HPV‐based screening compared to cytology‐based screening (RR 2.99, 95% 2.93–3.05) and colposcopies derived from the screening program were also more common (RR 1.68, 95% 1.63–1.73). p16/Ki67 triage resulted in more colposcopies (RR 1.86, 95% 1.76–1.95) than HPV16/18 (RR 1.54, 95% 1.44–1.65) and HPV16/18/31/33/52 (RR 1.63, 95% 1.55–1.71). The excess in colposcopy referrals was reduced when non‐screening‐derived colposcopies were included (intention‐to‐treat). Nevertheless, more women with CIN2 or worse were detected in the HPV group than in the cytology group per screened woman; in the p16/Ki67 triage group (RR 1.65, 95% 1.54–1.77), in the HPV16/18 group (RR 1.36, 95% 1.23–1.50), and in the HPV16/18/31/33/52 group (RR 1.48, 95% 1.37–1.59). HPV‐based screening, as compared with cytology screening, resulted in more screen positives, but all three triage algorithms substantially reduced the excess number of referrals to colposcopy. p16/Ki67 compared to triage with HPV16/18 may detect more cervical lesions.

## Graphical abstract

What's new?

Primary human papillomavirus (HPV)–based screening has shown superiority to cytology‐based screening in reducing cervical cancer risk in clinical trials. However, the benefit must be balanced with potential overdiagnosis/overtreatment. In this Danish health care policy trial, alongside the increased detection of women with underlying disease, HPV‐based screening resulted in two to three times more screen‐positive samples compared to well‐established cytology screening. Two triage algorithms using different thresholds of HPV genotypes and one with p16/Ki67 dual staining substantially reduced the excess number of referrals to colposcopy in HPV‐positive women with low‐grade cytological abnormalities, with p16/Ki67 detecting more cervical lesions.

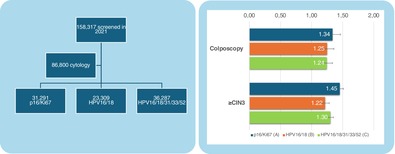

**Abbreviations**
CIN: cervical intraepithelial neoplasia
HCP: health care policy
HPV: human papillomavirus
ITT: intention to treat
NSLS: National Steering Committee for cervical cancer screening
p16/Ki67: staining for tumor suppressor, p16, and Ki‐67, a cellular marker for proliferation
RR: relative risk

## 1 INTRODUCTION

Evidence from clinical trials has shown that primary human papillomavirus (HPV)–based screening is superior to cytology‐based screening in reducing cervical cancer risk.^1^ The drawback of HPV‐based screening is that HPV infections are more common than cytology abnormalities, resulting in an increased number of HPV‐based screening positive women generating increased re‐test and gynecologist referrals.^1^ To reduce the proportion of women referred for follow‐up in HPV‐based screening based on HPV positivity alone, supplementary strategies using detection of HPV genotypes, cytology abnormalities, and molecular markers of HPV‐induced cellular changes can be used to triage to either re‐test or colposcopy. HPV‐based screening—including supplementary strategies—must balance the benefit of preventing cervical cancer against the potential harms of screening: overdiagnosis and overtreatment. Therefore, several countries have introduced HPV‐based screening as health care policy (HCP) trials aimed at ensuring the optimal ratio between prevented cancers and unnecessary re‐tests or referrals to gynecologist^2^, ^3^ in a “real life” setting.

In 2018, the Danish Health Authority mandated Danish regions to conduct a HCP trial of HPV‐based screening among women aged 30–59 in Denmark^4^ to ensure that HPV‐based screening would perform well in a Danish setting and to give the laboratories time to transition. As cervical cancer screening programs target entire populations with “one‐size‐fits‐all screening” as an entry point, the choice of triage becomes pivotal in securing an optimal balance between sensitivity of detection and specificity. Therefore, and as part of the HCP trial, the triage question was addressed by introducing three distinct triage options for HPV‐positive screening samples; liquid based cytology in combination with either p16/Ki67 dual staining, partial genotyping for HPV16, HPV18 or “other 12 high‐risk HPV genotypes” in bulk,^5^, ^6^ or extended genotyping with subdifferentiation between HPV genotypes HPV16, 18, 31, 33, 52 and HPV35, 39, 45, 51, 56, 59, 66, and 68.^7^, ^8^

The three triage strategies represent different approaches to triage. First, dual stain for p16/Ki67: A cytology slide is immunohistochemically stained for p16^INK4a^ and Ki67, markers indicative of an increased risk of underlying disease. Second, partial HPV16/18 genotyping: This method separates HPV16, HPV18, and other high‐risk HPV genotypes in combination with cytology as a triage strategy for HPV‐positive screening samples. This approach is well‐established and used in cervical cancer screening programs in countries such as the Netherlands, Australia, the USA, and Sweden. Third, extended genotyping and cytology: This strategy utilizes the differential risk associated with individual HPV genotypes to better identify women at increased risk of disease.

In the Danish HCP trial, we allocated one of the three triage algorithms per Danish region. In this paper we report the performance of the three algorithms in terms of their ability to identify high‐grade cervical lesions and reduce unnecessary referrals for re‐test or colposcopy. No HCP trial to date has systematically evaluated three HPV screening triage algorithms in a nationwide routine cervical cancer screening program.

## 2 METHODS

### 2.1 Study setting

The Danish cervical cancer screening program is a free‐of‐charge public health program operated by the five Danish regions, governed by national guidelines issued by the Danish Health Authority. The cervical cancer screening program is operated by public laboratories. Women aged 23–64 years are invited for screening participation by letter of invitation in a call‐recall program. The attendance has been slowly dropping within the last years and currently 61% of Danish women have a cervix cytology within 365 days from their invitation letter.^9^

This HCP trial was carried out embedded into the national routine screening program. The protocol was published on the National Steering Committee for cervical cancer screening (NSLS) website in January 2021^10^ and was later published in Danish.^4^ Women born on uneven days were allocated to HPV‐based screening and women born on even days were allocated to cytology‐based screening, which was the standard screening prior to the initiation of the study. All HPV‐positive screening samples were supplemented by liquid‐based cytology (from here referred to as cytology) and an additional triage. p16/Ki67 dual staining was used in two regions, two regions used cytology/partial HPV genotyping HPV16, HPV18, other HR, and one region used cytology and extended genotyping (HPV16, 18, 31, 33, 52, and other types), representing 43%, 22%, and 35% of the national screening activity, respectively.^9^

### 2.2 Study population, allocation, and data reporting period

Data were extracted in November 2023 from the Danish National Pathology Database which contains the result of all Danish cytology and histology samples. All Danish pathology departments report to the database and it is considered complete since 1999.^11^ All cytotechnicians undergo certification and in 2021 the recommendation was that pathology departments should analyze at least 25,000 samples per year.^9^

We included all women between 30 and 59 years of age with at least one sample registered in the national Patobank between the 4th of January and the 31st of December 2021. Only women with a screening sample were included (index sample), excluding women with any histological abnormality within the last 2 years, any cytological sample within the last 1.5 years, or if the current sample is follow‐up to a conization performed within the last 5 years. Women from the municipalities in Region of Southern Denmark, where primary HPV‐screening had been introduced as a clinical trial in 2017, were excluded.^12^ In this study, women with HPV16/18 were referred directly to colposcopy and cytology triage was carried out for women testing positive for other HPV types. Any cytological abnormality would lead to referral to colposcopy, resulting in a threefold increase in referral to colposcopy. In the present analysis, we included all follow‐up samples within 18 months from the index sample for each woman. See previous publication for complete description of study population and allocation.^13^

### 2.3 Triage methods and algorithm design

**FIGURE 1:**
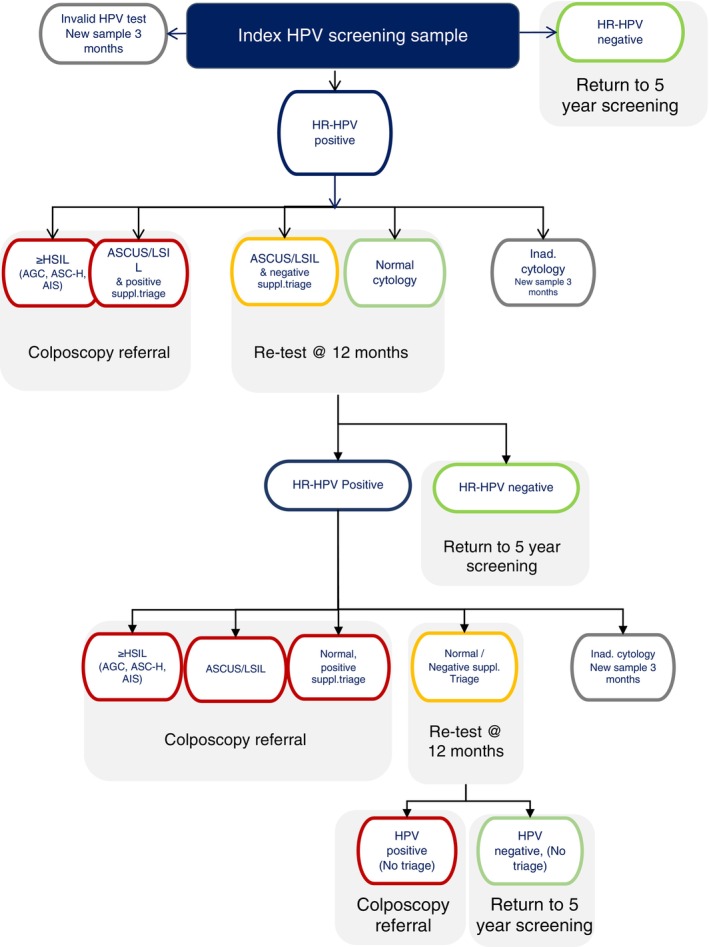
Flowchart for HPV group.

All HPV‐negative women were scheduled for a new screening after 5 years. Women with an HPV‐positive and high‐grade cytological abnormalities were referred directly to a gynecologist regardless of additional triage outcome (Figure 1). Women with a positive HPV test and low‐grade cytological abnormalities were managed according to the additional triage; p16/Ki67 dual‐staining, HPV16 or HPV18 positive, or HPV‐positive for either of HPV16, 18, 31, 33, 52 (Figure 1). Women positive by these criteria were referred for colposcopy, whereas those negative were referred for re‐testing in 12 months. Women with an HPV‐positive test and normal cytology were referred for re‐testing in 12 months regardless of additional triage outcome.

The HPV flowchart (Figure 1) covers a total of 24 months potential follow‐up time, at which point persistent HPV‐positive women will be referred to colposcopy regardless of triage. A second sample from a woman that was initially referred to re‐test will be categorized as a 12‐month sample even though the time between the samples is less—or more—than 12 months. A third sample from a woman that was referred to re‐test at first and second samples will be categorized as a 24‐month test.

**FIGURE 2:**
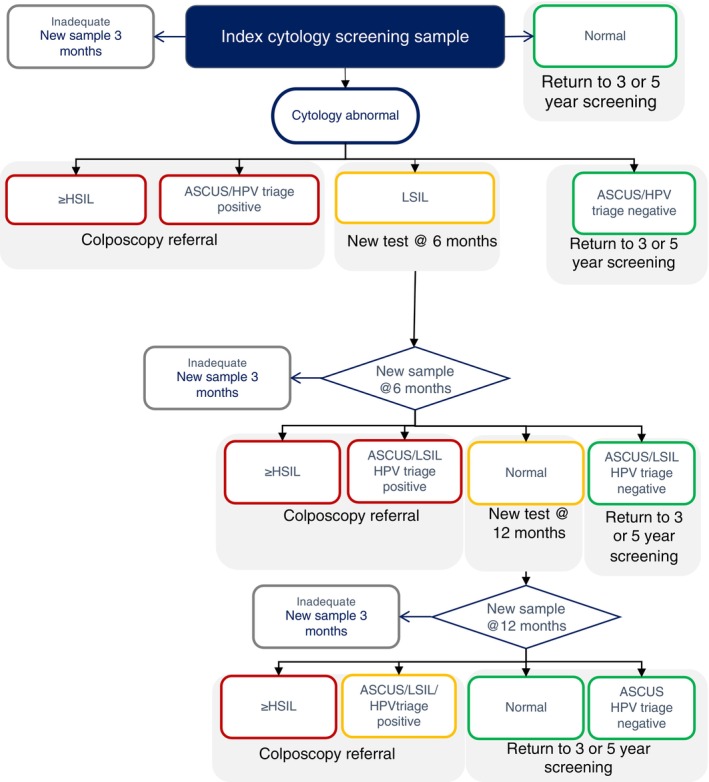
Flowchart for cytology group.

Women born on even days were allocated to screening with cytology, which was the standard screening prior to January 2021. Women with ASCUS were triaged with an HPV test. If this was positive, they would be referred to a gynecologist, and if it was negative, they would return to the screening program. Women with LSIL were recommended to have a repeat test after 6 months. See Figure 2 for further details.

All screening samples included in this study were collected in SurePath medium (BD Diagnostic, Sparks, MD) using a combi‐brush, spatula, or endocervical brush, and handled according to manufacturers’ instructions. For HPV testing the Central Denmark Region and Region of Southern Denmark used the Cobas 4800 HPV test, Region Zealand used the Cobas 6800 HPV test (both Roche Diagnostic, Pleasanton, CA). The Capital Region of Denmark and the North Denmark Region used the Onclarity HPV test (BD diagnostics, Sparks, MD). The Cobas tests detect 14 hrHPV types; HPV16 and HPV18 individually, whereas HPV31, 33, 35, 39, 45, 51, 52, 56, 58, 59, 66, and 68 reported jointly as “other HPV types.” The BD Onclarity HPV test reports HPV16, 18, 45, 31, 51, and 52 individually, combined with three grouped results for HPV33, 58, 35, 39, 68, and 56, 59, 66. The CINtec®plus immunohistochemistry test (Roche Molecular Systems, AZ) detects p16 and Ki67 proteins that are considered mutually exclusive in normal cells. The detection of one dual stained cell was considered as positive.

In Denmark, an examination by a gynecologist following a positive screening test includes colposcopy, cervical biopsy and endocervical curettage/cell‐sampling. Therefore, colposcopy and biopsies are used as interchangeable terms throughout the manuscript. For histology, the graded cervical intraepithelial neoplasia (CIN) classification was used (CIN1, CIN2, CIN3, adenocarcinoma in situ [AIS]) and carcinoma was classified according to WHO 2020 edition. In the category ≥CIN2 we include CIN2, CIN3, AIS, carcinoma, and tumors that meet the general criteria for classification as squamous cell carcinoma, but do not show sufficient distinctive features to be included in a more specific subtype, were also included as NOS (Not Otherwise Specified). The category ≥CIN3 we include, CIN3, AIS, and carcinoma. In cases with multiple histology outcomes for a woman, the most severe diagnosis was used.

### 2.4 Outcomes

For each of the three triage algorithms, we report number of screened women, number of women with a positive index sample, number of women referred to follow‐up, and number of women examined by a gynecologist. When possible, outcomes are reported in an intention‐to‐treat (ITT) approach, that is, a woman would not change group if she accidently received a different screening test than the one, she was allocated to. Visit to gynecologist would be included regardless of whether it was recommended or not.

### 2.5 Data retrieval and statistical method

All five regions initiated the trial on the 4th of January 2021. The first registered screening sample during the recruitment period was defined as the index sample. Referral was defined as the action recommended based on the combined results of the index sample and the triage. The histology outcome of the screening was defined as the most severe histology diagnosis observed within 18 months of the index sample, irrespective of whether this histology was performed. Register‐based follow‐up was conducted for all outcomes for each woman during the first 18 months following her index sample, with the last data retrieval undertaken in November 2023. Data were retrieved on behalf of the NSLS by the Danish Regions Clinical Quality Development Program. Relative risk of the different outcomes as well as 95% confidence intervals using logarithmic transformation^14^ was calculated using Excel.

Data from each triage algorithm was compared with the same region cytology data to eliminate regional differences in cytology procedures and interpretation. Relative risk ratios were used to compare outcomes of the triage algorithms. The report follows CONSORT reporting guideline (Data S1, Supporting Information).^15^

## 3 RESULTS

**FIGURE 3:**
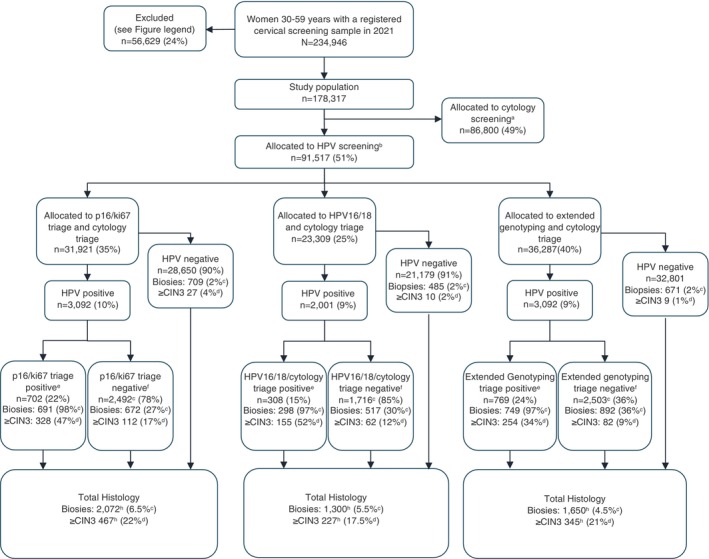
Flow and histology results of the three HPV triage algorithms. Percentages are out of the total amount of women of the box above unless anything else is specified. For simplicity, insufficient samples, wrong codes, and wrong tests are not reported in the flowchart. Extended genotyping includes HPV16, 18, 31, 33, and 52. Excluded women: first cytology after conization 2274, second cytology after conization and previously recommended follow‐up 3018, previous cytology recommended follow‐up 13,064, previous histological abnormality 1110, any cytology within 1.5 year 10,924, samples from Southern Region that were already screened with HPV as part of pilot 26,239. Superscript letters: a, women born on even days; b, women born on uneven days; c, out of all women in current box; d, out of all biopsies; e, triage positives are women with HSIL or LSIL/ASCUS with a positive supplementary triage (either positive for p16/Ki67, HPV16/18 or extended genotyping). Triage positive women are referred to biopsies; f, triage negatives are women with normal cytology or LSIL/ASCUS with negative supplementary triage. Triage negative women are referred to a retest in 12 months; g, include insufficient samples and the total across the two boxes are therefore higher than the above total; h, totals are lower than in Table 1 because biopsies from women with insufficient samples, wrong codes, or wrong handlings are not counted in this flow diagram.

We included 178,317 Danish women between 30 and 59 years of age that had a registered screening sample in 2021 after the municipalities of the Southern region—that had previously introduced HPV testing—were excluded (Figure 3). Around half of the screened women (49%) were born on an even day and therefore allocated to cytology. Another 51% were born on an uneven day and therefore screened with cytology. A total of 31,921 (35%) of the HPV samples were registered in the two regions using p16/ki67 triage, 23,309 (25%) in the two regions using partial HPV genotyping triage, and 36,287 (40%) samples in the region using extended genotyping triage. Detailed flow diagram for all six groups can be seen in Figure S1.

Overall, 9.1% of the women were HPV‐positive, compared to 3.0% among the cytology‐screened women (Table 1). Regions using p16/Ki67 triage had 9.7% women testing positive for HPV, regions using partial genotyping had 8.6% screening positive, whereas the region using extended genotyping had 9.0% screening positive (Table 1). After triage, between 1 and 3% of all women were referred to colposcopy (Table 1). p16/Ki67 triage resulted in slightly more initial referrals to colposcopy compared to cytology (RR = 1.11, 95% CI 1.00–1.24). In contrast, partial genotyping and extended genotyping resulted in slightly fewer initial referrals compared to cytology (RR = 0.85, 95% CI 0.73–0.99 and RR = 0.82, 95% CI 0.74–0.90).

**TABLE 1:** Outcome of screening within 18 months in women with an index sample taken between 1st of January 2021 and 31st of December 2021 by triage algorithm.

	Regions with HPV screening and p16/Ki67 triage			Regions with HPV screening and triage with partial (HPV16/18) genotyping			Regions with HPV screening and triage with extended genotyping (HPV16/18/31/33/52)			All regions		
	HPV^a^	Cytology^a^	Relative risk (95% CI)	HPV^a^	Cytology^a^	Relative risk (95% CI)	HPV^a^	Cytology^a^	Relative risk (95% CI)	HPV^a^	Cytology^a^	Relative risk (95% CI)
Screened	31,921 (100)	30,202 (100)	‐	23,309 (100)	22,312 (100)	‐	36,287 (100)	34,286 (100)	‐	91,517 (100)	86,800 (100)	‐
Screened positive	3092 (9.7)	785 (2.6)	3.73 (3.60–3.85)	2001 (8.6)	748 (3.4)	2.56 (2.46–2.67)	3248 (9.0)	1113 (3.2)	2.76 (2.67–2.85)	8341 (9.1)	2646 (3.0)	2.99 (2.93–3.05)
Insufficient	102 (0.3)	259 (0.9)	0.37 (0.31–0.45)	23 (0.1)	217 (1.0)	0.10 (0.07–0.15)	24 (0.1)	192 (0.6)	0.12 (0.08–0.18)	149 (0.2)	668 (0.8)	0.21 (0.18–0.25)
Others^b^	77 (0.2)	13 (0.0)	‐	106 (0.5)	7 (0.0)	‐	124 (0.3)	5 (0.0)	‐	307 (0.3)	25 (0.0)	‐
% of screened women												
Referred to colposcopy	702 (2.2)	599 (2.0)	1.11 (1.00–1.24)	308 (1.3)	348 (1.6)	0.85 (0.73–0.99)	769 (2.1)	891 (2.6)	0.82 (0.74–0.90)	1779 (2.0)	1838 (2.0)	0.92 (0.86–0.98)
Referred to retest^c^	2492 (7.8)	445 (1.5)	5.30 (4.80–5.75)	1716 (7.4)	617 (2.8)	2.66 (2.43–2.91)	2503 (6.9)	414 (1.2)	5.71 (5.15–6.33)	6711 (7.3)	1476 (1.7)	4.31 (4.08–4.56)
Back to screen	28,650 (89.8)	29,145 (96.5)	0.93 (0.93–0.94)	21,179 (90.9)	21,340 (95.6)	0.95 (0.95–0.96)	32,891 (90.6)	32,976 (96.2)	0.95 (0.94–0.95)	82,720 (90.8)	83,461 (96.2)	0.94 (0.94–0.95)
Initially referred to colposcopy												
Had colposcopy	691 (2.2)	575 (1.9)	1.14 (1.06–1.22)	298 (1.3)	330 (1.5)	0.86 (0.77–0.97)	749 (2.1)	858 (2.5)	0.82 (0.77–0.89)	1738 (1.9)	1763 (2.0)	0.94 (0.89–0.98)
% of women with colposcopy												
≥CIN2	462 (66.8)	323 (56.2)	1.19 (1.07–1.32)	214 (71.8)	187 (56.7)	1.27 (1.10–1.46)	406 (54.2)	379 (44.2)	1.23 (1.12–1.34)	1082 (62.3)	889 (50.4)	1.23 (1.16–1.31)
≥CIN3	328 (47.5)	246 (42.8)	1.11 (1.00–1.23)	155 (52.0)	140 (42.4)	1.23 (1.06–1.41)	254 (33.9)	229 (26.7)	1.27 (1.14–1.42)	737 (42.4)	615 (34.9)	1.22 (1.14–1.30)
Combined result of women initially referred to either colposcopy or retest												
Had colposcopy	1363 (4.3)	695 (2.3)	1.86 (1.76–1.95)	815 (3.5)	507 (2.3)	1.54 (1.44–1.65)	1641 (4.5)	952 (2.8)	1.63 (1.55–1.71)	3819 (4.2)	2154 (2.5)	1.68 (1.63–1.73)
% of women with colposcopy												
≥CIN2	673 (49.4)	363 (52.2)	0.95 (0.86–1.04)	350 (42.9)	268 (47.1)	0.91 (0.81–1.02)	644 (39.2)	396 (41.6)	0.94 (0.87–1.02)	1667 (43.7)	998 (46.3)	0.94 (0.90–0.99)
≥CIN3	440 (32.3)	273 (39.3)	0.82 (0.75–0.91)	217 (26.6)	166 (32.7)	0.81 (0.71–0.93)	336 (20.5)	232 (24.4)	0.84 (0.76–0.93)	993 (26.0)	671 (31.2)	0.83 (0.79–0.89)
All colposcopies (women initially referred to either colposcopy, retest or back to screen, intension‐to‐treat)												
Had colposcopy	2089 (6.5)	1480 (4.9)	1.34 (1.28–1.39)	1328 (5.7)	1018 (4.6)	1.25 (1.19–1.32)	2337 (6.4)	1775 (5.2)	1.24 (1.20–1.29)	5754 (6.3)	4273 (4.9)	1.28 (1.25–1.31)
% of women with colposcopy												
≥CIN2	717 (34.3)	411 (27.8)	1.24 (1.16–1.32)	374 (28.2)	264 (25.9)	1.09 (0.99–1.19)	664 (28.4)	425 (23.9)	1.19 (1.11–1.27)	1755 (30.5)	1199 (25.7)	1.18 (1.14–1.24)
≥CIN3	474 (22.7)	307 (20.7)	1.09 (1.01–1.19)	235 (17.7)	185 (18.2)	0.97 (0.86–1.10)	348 (14.9)	253 (14.3)	1.04 (0.95–1.15)	1057 (18.4)	745 (17.4)	1.05 (1.00–1.11)

*Note*: Relative risk (RR) and 95% confidence interval (CI) of HPV‐screening compared with cytology‐screening in the same region.

^a^ Numbers in parenthesis are percentages.

^b^ Misclassifications according to allocation.

^c^ Include insufficient samples.

A total of 7.3% of the women in the three HPV groups were referred to re‐testing (HPV‐positive with normal cytology or HPV‐positive with ASCUS/LSIL and negative triage) compared to 1.7% (women with LSIL) in the cytology group. Compared to cytology, the relative risk of re‐testing was lower with partial genotyping triage (RR = 2.66) compared to p16/Ki67 (RR = 5.30) and extended genotyping (RR = 5.71). The difference is partly explained by a higher referral rate for re‐testing in the cytology group in the two regions using HPV16/18 as triage (2.8% vs. 1.2% and 1.5%). Fewer women were returned to screening in the HPV group compared to the cytology group with no major differences between the three triage algorithms. Triage of low‐grade cytological abnormalities was positive in 67.1% of cases when using p16/Ki67, 21.7% of cases when using partial genotyping, and in 52.8% of cases when using extended genotyping (Table S1).

Almost all women referred to colposcopy after index screening sample had a colposcopy as recommended (97%). Significantly more ≥CIN2 and ≥CIN3 per colposcopy were found in HPV‐based screening over cytology (RR = 1.23, 95% CI 1.16–1.31 and RR = 1.22, 95% CI 1.14–1.30; Table 1). No significant difference between the three triage algorithms was observed.

Adherence to follow‐up recommendation for re‐testing in 12 months was high in all three triage algorithms, with 84–88% having a registered re‐test within the follow‐up period. At the 12 months re‐testing, more women were referred to colposcopy in the extended genotype triage (34%) compared to the other triage algorithms (22 and 28%, respectively; Table 1). However, extended genotyping triage resulted in very few referrals to 24 months re‐testing (16% compared to 26 and 33% in the other two triage algorithms). As expected with an 18‐month follow‐up period, the 2nd re‐testing compliance was low at the time of data retrieval. Only 9–15% had undergone this third test, with 1300 women still awaiting this final re‐test at the time of data retrieval.

When colposcopies after index screening sample were combined with the colposcopies performed after 12 months re‐testing, referral for colposcopy increased in HPV‐based screening compared to cytology screening (RR = 1.68, 95% CI 1.63–1.73; Table 1). The colposcopy referral difference was smallest in the partial HPV16/18 genotyping triage algorithm (RR = 1.54, 95% CI 1.44–1.65; Table 1) compared with the p16/Ki67 triage or extended genotyping. Each colposcopy after HPV‐based screening was slightly less likely to find ≥CIN2 (RR = 0.94, 95% CI 0.90–0.99; Table 1) compared to cytology screening with no differences between the three triage algorithms. The pattern for ≥CIN3 was similar.

Many colposcopies were performed among women that were recommended to return‐to‐screening after a negative index test (see Figure S2). For HPV‐based screening, an additional 1935 colposcopies were done and for cytology screening, 2119 colposcopies were done. When we include all colposcopies in an intention‐to‐treat analysis, still more colposcopies were conducted in the HPV‐based screening (RR = 1.28, 95% CI 1.25–1.31; Table 1) compared to cytology, and with no differences among the three triage algorithms (Table 1). More ≥CIN2 was found per colposcopy in HPV‐based screening (RR = 1.18, 95% CI 1.14–1.24; Table 1) compared to cytology. Between triage algorithms, there was a tendency of finding more ≥CIN2 in HPV‐screened women triaged with p16/Ki67 compared to partial HPV16/18 genotyping or extended, but with overlapping confidence intervals (Table 1). Among all ITT colposcopies, there was a tendency toward higher detection of ≥CIN3 per colposcopy when using p16/Ki67 or extended genotyping triage, whereas partial genotyping was at par/less than in cytology‐screened women (Table 1). Due to higher rates of colposcopies among HPV‐screened women compared to cytology‐screened women, more ≥CIN3 was found per screened woman in the HPV group (RR = 1.34, 95% CI 1.26–1.43, not tabulated).

**FIGURE 4:**
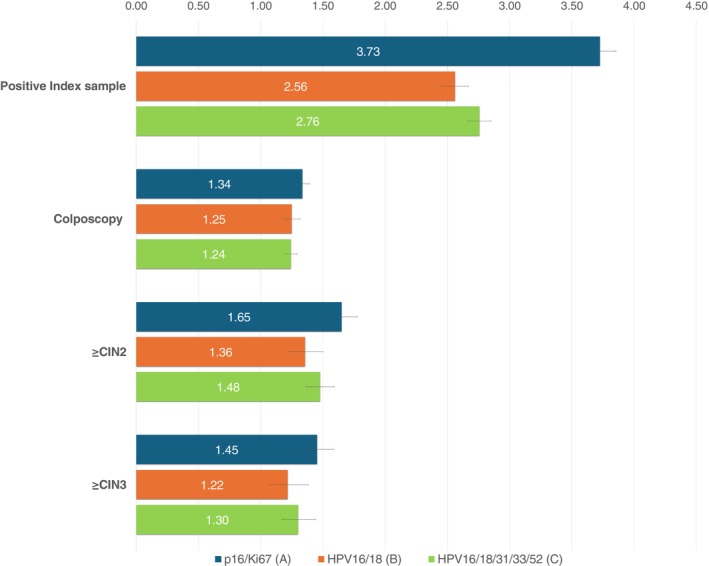
Shows relative risk for each triage algorithm compared to cytology in the same regions out of all women screened. 95% confidence intervals are marked as black lines. Intention‐to‐treat data.

Figure 4 shows the relative outcomes per screened woman for the three different triage algorithms. The relative risk of a positive index sample was higher for p16/Ki67 than for the other algorithms compared to cytology. However, this difference stems from the number of positive cytology index samples between the different regions (ranging from 2.6% in the regions with p16/Ki67 to 3.4% in the regions that use HPV16/18 triage). The relative risk of colposcopies compared to cytology is slightly higher in the regions with p16/Ki67 triage but with overlapping confidence intervals. The regions with p16/Ki67 appear to find relatively more ≥CIN2, again compared to the paired cytology screening (RR = 1.65, 95% CI 1.54–1.77; Figure 4) compared to regions with HPV16/18 (RR = 1.36, 95% CI 1.23–1.50).

## 4 DISCUSSION

We found that newly implemented HPV‐based screening resulted in two to three times more screen‐positive samples compared to the well‐established cytology screening, and that HPV‐based screening also resulted in an increased detection of women with underlying disease. These results are in accordance with other HCP trials.^2^ After the HPV index screening round, the number of women referred to colposcopy was almost doubled for p16/Ki67 triage; 50% increased for partial genotyping, and 60% increased for extended genotype triage, when compared to the regionally cytology screening. Including all ITT colposcopies, the number of extra colposcopies went down to 20–30% more compared to cytology, with no difference observed between the three HPV‐triage algorithms. Detection of ≥CIN2 was increased by 65% for p16/Ki67 and by approximately 40% for both partial and extended genotyping. Detection of ≥CIN3 increased by 45% for p16/Ki67 and by 20–30% for the partial and extended genotyping.

The Danish HCP trial has the advantage of comparing different triage algorithms within routine national screening, which distinguishes these results from most studies that only evaluate one HPV triage modality against existing cytology. Furthermore, previous studies included only a few thousand women, whereas our study involved over 30,000 women and included a control group.

Comparing the individual triage algorithms, we expected to find more colposcopies in the region using extended genotyping triage compared to partial genotyping, which was also the case measured on colposcopy referrals after the index screening round. However, in the intention‐to‐screen analysis this difference disappeared due to many colposcopies among index screened negative women. Although the indication for biopsies is not registered in the Danish registries a possible explanation could be that the biopsies were taken because the women had symptoms. In 2021 all regions in Denmark recommended that women with postcoital bleeding had colposcopy along with biopsies.

Due to the high‐risk nature of HPV16 and HPV18 infections,^16^, ^17^ we expected to find more ≥CIN2 per colposcopy in the regions using partial HPV16/18 genotyping than in the regions using extended genotyping, but this was not the case. A possible explanation could be that we only referred women directly to colposcopy that had a concurrent ASCUS/LSIL, whereas women with HPV16/18 and normal cytology were referred to re‐testing. Extended genotyping offers potential to refine management of patients with specific HPV genotypes based on risk of disease,^8^, ^16^ this option is not available using partial genotyping as the remaining 11 carcinogenic HPV genotypes are indistinctively reported as a bulk “yes/no” outcome. A recent Swedish study recommends HPV16 and 18 referral to colposcopy regardless of cytology triage.^18^ However, our HCP trial shows that this would increase the initial number of colposcopies when substituting cytology screening with HPV‐based screening.

A recent review^19^ summarized the evidence on triage algorithms for HPV‐positive women. Many studies have examined p16/Ki67 staining triage of HPV‐positive women compared to cytology triage and found varying diagnostic accuracy.^20^, ^21^, ^22^, ^23^ In our study, p16/Ki67 staining was performed concurrently with cytology triage, but without information on specific genotype, and compared to cytology screening. Our results indicated that a significant increase in colposcopy referrals would ensue if the group of HPV‐positive, p16/Ki67‐positive women with normal cytology triage were referred for colposcopy instead of our chosen 1 year re‐test interval.

A study from the Scottish screening program calculated the highest sensitivity of a three‐step approach where HPV16/18 were considered positive and other high‐risk types would have reflex cytology and be considered positive in case of abnormalities.^24^ The women with negative reflex cytology had p16/Ki67 staining. A Canadian randomized controlled trial compared HPV test with reflex cytology to cytology, and found an almost 90% increase in colposcopies in the HPV group.^25^ We found a similar increase in colposcopies for HPV‐screened women with p16/Ki67 and extended genotyping when colposcopies performed on women who tested negative at the index screen were excluded. In our study we used a supplementary triage in addition to cytology triage, and we would therefore expect fewer colposcopies than in the Canadian study, but after including colposcopies after referral/re‐testing our proportions were similar to that of the Canadian study. In a future study we will explore why our supplementary triage did not reduce the number of colposcopies. The Canadian study found a 60% increase in ≥CIN2 which is close to the 36–65% increase found in our study (Figure 4).

A recent cost effectiveness analysis compared p16/Ki67 triage to HPV genotyping and concluded that adding p16/Ki67 would increase cost, but since it was expected to reduce disease it was considered cost effective.^26^ In our study we also found that more cervical lesions were found with p16/Ki67 triage, and this could possibly reduce the risk of cervical cancer. However, from an operational perspective, p16/Ki67 staining requires operator training and is subjective compared to use of HPV genotyping, whether partial or extended.^27^

The strength of this study was its size and the integration into a national screening program, and therefore shows how screening recommendations work in “real life” setting. A limitation of this study was differences in referrals to colposcopy and referrals to re‐testing in the cytology groups, which indicated local differences in disease burden and/or diagnostic practices. However, the study was controlled and had an internal reference group which should make the groups comparable. Our follow‐up was relatively short which made it difficult to calculate meaningful sensitivities and specificities. We are lacking follow‐up data on women in the HPV algorithms referred for more than 18 months of follow‐up. If these data were included, it would lead to more disease found in the HPV group. However, since women undergoing repeated retesting are probably at lower risk for cervical lesions than women referred after a positive index sample (highest risk group), the balance between detected cervical lesions and the number of colposcopies might not be as favorable as suggested by the presented results. Since cytology is less sensitive, it is expected that more disease would be found in a second round of cytology screening compared to HPV screening, which makes comparison complicated. Given the large number of colposcopies among the women that initially tested negative, we believe that it would only change our results slightly. We anticipate that the increased risk of positive screening samples and colposcopies will be counteracted by the fact that women in the HPV group are recommended a new screening every 5 years compared to every 3 years in the cytology group.

## 5 CONCLUSION

The first round of HPV‐based screening, compared with the long‐standing cytology screening, resulted in more screen‐positive cases and more referrals to colposcopies; however, these numbers were substantially reduced by the three triage algorithms. The three triage algorithms resulted in similar outcomes, but we found slightly more severe and moderate cervical lesions with p16/Ki67 compared to partial and extended genotyping.

## AUTHOR CONTRIBUTIONS

**Jeppe Bennekou Schroll:** Conceptualization; methodology; validation; software; data curation; writing – original draft; investigation; writing – review and editing; visualization. **Jesper Bonde:** Conceptualization; methodology; validation; investigation; writing – review and editing; resources; data curation. **Elsebeth Lynge:** Conceptualization; methodology; validation; writing – review and editing; formal analysis; data curation. **Marianne Waldstrøm:** Writing – review and editing; conceptualization; methodology; validation; formal analysis; resources. **Petra Hall Viborg:** Software; validation; writing – review and editing; data curation. **Anna Frandsen:** Resources; writing – review and editing. **Rikke Holst Andersen:** Writing – review and editing. **Susanne Merete Nielsen:** Writing – review and editing. **Bettina Kjær Kristensen:** Project administration; writing – review and editing; validation; conceptualization; methodology; investigation; formal analysis; data curation. **Doris Schledermann:** Writing – review and editing. **Berit Andersen:** Writing – review and editing; resources; conceptualization; methodology; validation; formal analysis; data curation.

## CONFLICT OF INTEREST STATEMENT

JB: Institution has received research funding and/or consumables at reduced price or for free to support research from BD Diagnostics (US) and Seegene (South Korea). JB has received honoraria for lectures from BD Diagnostics. JBS, EL, BA, BKK, PHV, MW, DS, AF, RHA, SMN: No conflicts of interest.

## Supporting information


**Data S1.** Supporting Information.

## Data Availability

Data can be made available through an application to the Danish Healthcare Quality Institute. Further information is available from the corresponding author upon request.
