# Supplementary material for: Health care policy trial of primary human papillomavirus–based cervical screening in Denmark: Comparison of three triage algorithms
Source: Int J Cancer. 2026 Feb 7;158(12):3197–207. doi: 10.1002/ijc.70365 (PMC13106925; doi:10.1002/ijc.70365)
Supplement: Supplementary file 1 — Data S1. Supporting Information. [file IJC-158-3197-s001.pdf]

Health care policy trial of primary human papillomavirus–based cervical screening in Denmark: Comparison of three triage algorithms

Jeppe Bennekou Schroll, Jesper Bonde, Elsebeth Lynge, Marianne Waldstrøm, Petra Hall Viborg, Anna Frandsen, Rikke Holst Andersen, Susanne Merete Nielsen, Bettina Kjær Kristensen, Doris Schledermann, Berit Andersen on behalf of the National Danish Cervical Cancer Screening Steering Committee

Table of contents

Supplementary Table 1. Result of initial triage of all HPV positive woman (only supplementary triage low-grade cytological abnormalities has clinical consequences) **2**

Supplementary Figure 1. Sankey plot of recommended referrals based on sample results. **3**

Supplementary Figure 2. Sankey plot of actual colposcopies grouped by index sample **4**

Supplementary Table 1. Result of initial triage of all HPV positive woman (only supplementary triage low-grade cytological abnormalities has clinical consequences)

	High grade cytological abnormalities		Low grade cytological abnormalities		Normal cytology		Total
	Supl. triage+	Supl. triage-	Supl. triage+	Supl. triage-	Supl. triage+	Supl. triage-	
P16/Ki67	360 (95.2)	18 (4.8)	324 (67.1)	159 (32.9)	581 (26.0)	1,650 (74.0)	2,001
Partial genotyping	103 (48.6)	109 (51.4)	310 (21.7)	277 (78.3)	1,123 (24.1)	1,079 (75.9)	3,248
Extended genotyping	325 (70.8)	134 (29.2)	310 (52.8)	277 (47.2)	1,123 (51.0)	1,079 (49.0)	3,092

Numbers in parenthesis is percentage of all women in that cervical cytological category.
High grade cytological abnormalities: ASC-H/HSIL/AGC/AIS/carcinoma
Low grade cytological abnormalities: ASC-H/HSIL/AGC/AIS/carcinoma

Supplementary Figure 1. Sankey plot of recommended referrals based on sample results.

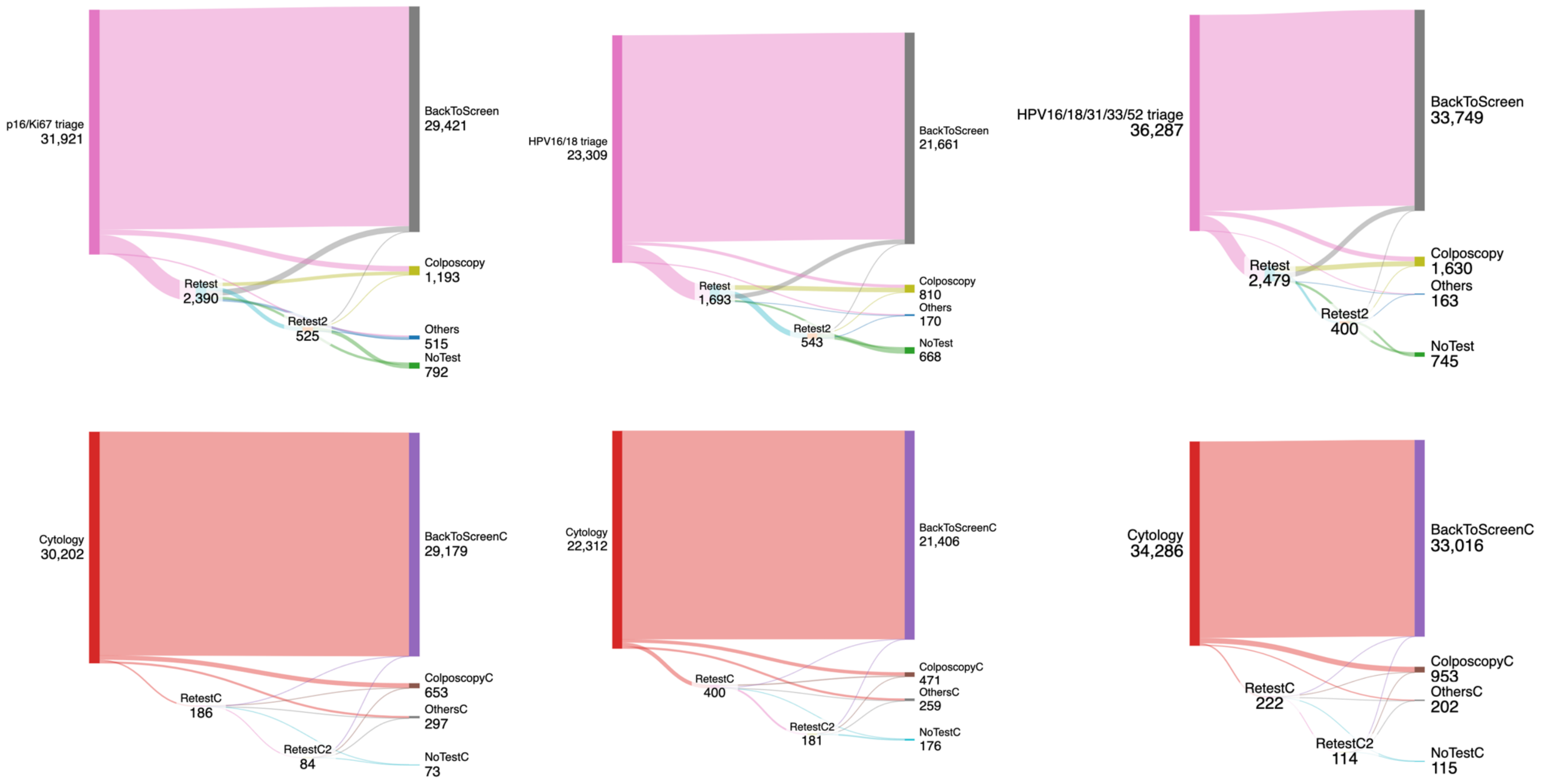

Others is a combination of misclassified samples and insufficient samples.

Supplementary Figure 2. Sankey plot of actual colposcopies grouped by index sample

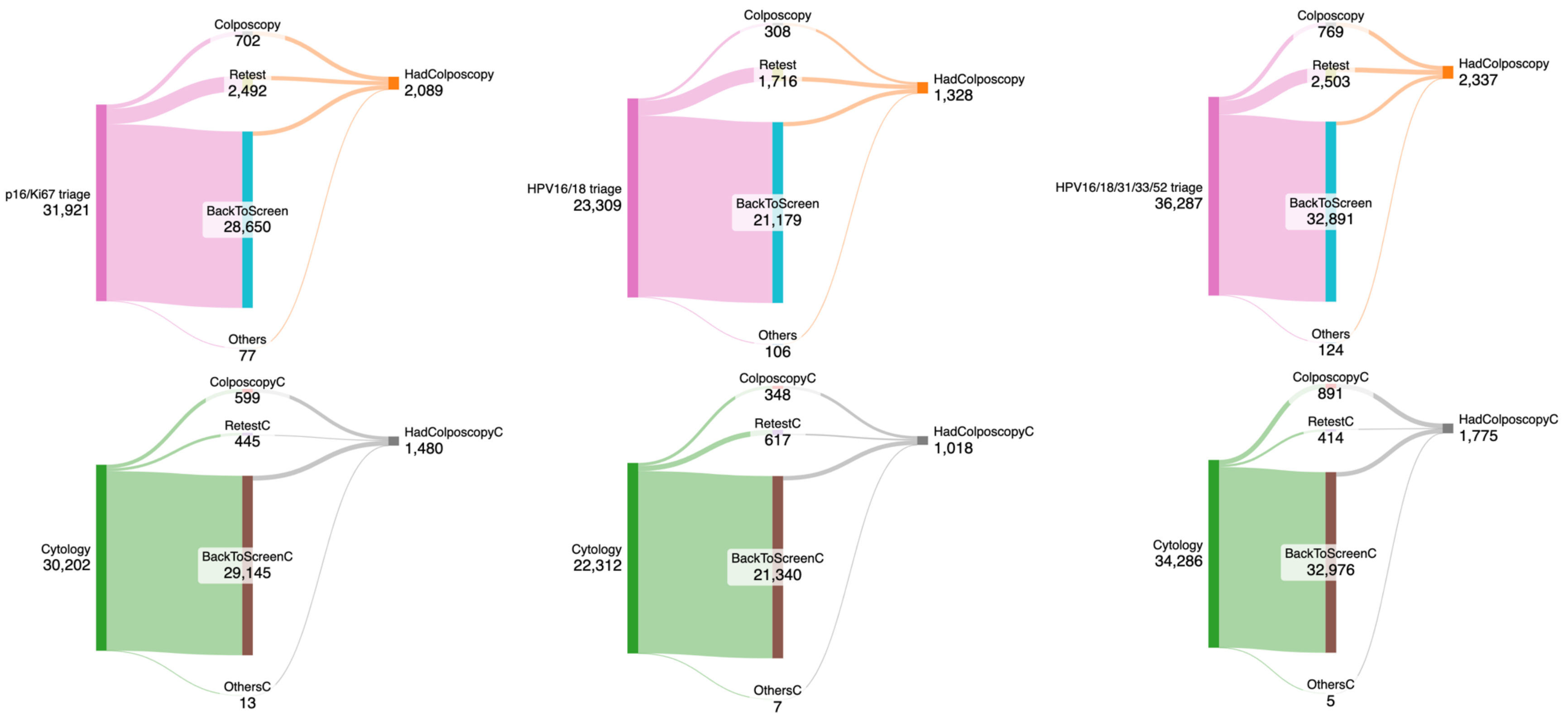

The retest group includes insufficient sam
